# Risk factors and immune landscape of early local tumor progression after microwave ablation for lung cancer: a retrospective nested case-control study

**DOI:** 10.3389/fimmu.2026.1767345

**Published:** 2026-03-06

**Authors:** Nan Wang, Jingwen Xu, Ji Ma, Siyi Niu, Xiuhong Ren, Qi Xie, Zhigang Wei, Xin Ye

**Affiliations:** 1 Shandong University of Traditional Chinese Medicine, Department of First Clinical Medical College, Shandong Provincial Qianfoshan Hospital, Jinan, China; 2 Department of Oncology, The First Affiliated Hospital of Shandong First Medical University & Shandong Provincial Qianfoshan Hospital, Shandong Lung Cancer Institute, Jinan, China; 3 Shandong Medicine and Health Key Laboratory of Cardiac Electrophysiology and Arrhythmia, The First Affiliated Hospital of Shandong First Medical University & Shandong Provincial Qianfoshan Hospital, Department of Cardiology, Jinan, China; 4 Shandong Provincial Lab for Clinical Immunology Translational Medicine in Universities, Jinan, China; 5 Cheeloo College of Medicine, Shandong University, Jinan, China

**Keywords:** immune microenvironment, incomplete microwave ablation, local tumor progression, lung cancer, risk factors

## Abstract

**Background and objectives:**

Microwave ablation (MWA) is an effective therapy for early-stage inoperable non-small cell lung cancer (NSCLC), yet its efficacy is limited by early local tumor progression (LTP). As early LTP is often suggestive of incomplete ablation, this study aimed to identify its risk factors and to characterize the associated changes in systemic immune parameters.

**Methods:**

This single-center retrospective nested case-control study enrolled patients with NSCLC who underwent MWA between January 1, 2021, and December 31, 2023. Patients were divided into an early LTP group (LTP ≤6 months post-MWA) and a control group. Clinical data and peripheral blood immune parameters at pre-MWA, one-week post-MWA, and one-month post-MWA were collected. Univariate and multivariate logistic regression analyses were used to identify independent risk factors and dynamic changes in immune indicators compared between groups.

**Results:**

A total of 76 patients were included (19, early LTP group; 57, control group). Multivariate analysis identified three independent risk factors for early LTP: maximum tumor diameter >30 mm (OR = 2.681, 95%CI: 1.218–5.901, P = 0.014), distance to hilum ≤10 mm (OR = 3.280, 95%CI: 1.678–6.411, P = 0.001), and ablative safety margin (≤5.0 mm) (OR = 4.152, 95%CI: 1.922–8.968, P < 0.001). Comparative analysis of peripheral blood immune parameters revealed distinct patterns between groups at one-month post-MWA. Compared to the control group, the early LTP group exhibited a significant reduction in CD4^+^ T cells (P = 0.040) and IL-2 levels (P = 0.020), whereas IL-10 (P < 0.001) and IL-6 (P = 0.004) levels were significantly elevated.

**Conclusion:**

Large tumor size, proximity to the pulmonary hilum, and an insufficient ablative safety margin are key risk factors for early LTP post-MWA. The development of early LTP is associated with significant alterations in specific peripheral blood immune cell subsets and cytokine levels at one-month post-MWA.

## 1 Introduction

Lung cancer is one of the malignant tumors with the highest global incidence and mortality rates (1). For early-stage lung cancer, anatomical resection remains the standard curative treatment; however, many patients are ineligible for surgery due to comorbidities or poor functional status (2, 3). Image-guided percutaneous thermal ablation techniques, such as microwave ablation (MWA), have become important treatment alternatives for patient’s ineligible for surgery (4, 5). MWA induces coagulative necrosis of tumor cells by generating frictional heat through high-frequency electromagnetic waves. Compared to radiofrequency ablation, MWA has faster ablation speed, larger ablation volume, and less susceptibility to heat-sink effects (6, 7). However, local tumor progression (LTP) due to incomplete microwave ablation (iMWA) remains a challenge. Currently, there is no established method for direct and immediate intraprocedural determination of iMWA. Consequently, in clinical practice and research, early LTP within 6 months post-MWA is widely regarded as a surrogate indicator suggestive of technically insufficient ablation (8–11). Beyond the inherent risk of residual tumor cells retaining proliferative capacity, the occurrence of early LTP may be closely associated with alterations in the host immune microenvironment (12, 13). Therefore, it is crucial to identify the risk factors predisposing to early LTP and to understand the subsequent changes in the host immune profile. Currently, most studies on risk factors for early LTP after ablation primarily focus on tumor anatomical characteristics and technical parameters (14, 15). In particular, the achievement of an adequate ablative safety margin is considered a critical determinant of success, yet a standardized, quantitative threshold for lung MWA remains to be established. Although thermal ablation is known to release tumor antigens, potentially activating systemic anti-tumor immunity and forming the basis of the abscopal effect (16, 17), this immune response is considered transient and complex, exhibiting phase-dependent and dual characteristics (18). Specifically, the interaction between the post-ablation state and the host immune system in the context of early LTP, and its associated systemic immune profile, remains unclear. Therefore, this retrospective nested case-control study aims to address two core questions: first, to identify clinical and procedural risk factors for early LTP after MWA for lung cancer; second, to characterize the dynamic changes in peripheral blood immune cell subsets and cytokine levels before and after ablation between patients with and without early LTP, thereby exploring the potential association between this outcome and systemic immune alterations.

## 2 Materials and methods

### 2.1 Study design and participants

This study was approved by the hospital’s ethics committee (Approval No.: XMSBLL2025(468)) who waived the need for informed consent. We retrospectively analyzed all patients with primary lung cancer who underwent CT-guided percutaneous pulmonary MWA at our institution between January 1, 2021, and December 31, 2023.

Inclusion criteria: ① Pathologically confirmed non-small cell lung cancer (NSCLC); ② Complete MWA procedure records and regular postoperative follow-up imaging data (chest CT reviews at 1-, 3-, 6-, and 12-months post-procedure), with follow-up ≥12 months; ③ Complete peripheral blood immunology test results available from pre-procedure, within one-week post-procedure, and at one-month post-procedure.

Exclusion criteria: ① Other concurrent active malignancies; ② Previous local treatment in the target lesion before ablation; ③ Any systemic therapy or condition during the perioperative period known to significantly alter immune status, including but not limited to immunotherapy, chemotherapy, systemic corticosteroid use, or acute inflammatory conditions; ④ Suffering from systemic diseases severely affecting immune function; ⑤ Incomplete clinical data.

### 2.2 Group definition and LTP assessment

This study adopted a retrospective nested case control design. The study population comprised 1,211 patients with non-small cell lung cancer who underwent MWA after meeting the inclusion and exclusion criteria. Patients were grouped according to follow-up outcomes: the early LTP group consisted of 19 patients who showed imaging-confirmed LTP within ≤6 months post-MWA. To enable efficient comparative analysis, capture all potential risk factors, and avoid preset limitations, a random sampling approach was used to select the control group from the 1,192 patients without evidence of LTP within 6 months post-MWA. Using a random number table, 57 controls were selected in a 1:3 case-to-control ratio.

LTP was assessed radiologically based on established post-MWA imaging criteria. It was defined as the appearance of a new or progressively enlarging nodular or irregular enhancing focus at the edge or within the immediate adjacent region (≤1 cm) of the ablation zone on contrast-enhanced CT. This definition aligns with the standard practice of monitoring the ablation zone and its periphery for signs of recurrence, such as abnormal nodular enhancement or enlargement (19), and incorporates a quantifiable distance threshold for operational consistency. Furthermore, the ≤1 cm boundary is clinically relevant as it corresponds to the ideal minimal “ablative safety margin” (10 mm) recommended for curative thermal ablation (20). Progression identified within this region is therefore highly indicative of an insufficient ablative margin from the initial procedure.

### 2.3 Data collection

The following data were collected from electronic medical records. ①Patient baseline characteristics: age, sex, Eastern Cooperative Oncology Group (ECOG) performance status. ②Tumor characteristics: pre-MWA CT-measured maximum tumor diameter, density (solid/subsolid), distance to the hilum and major blood vessels (diameter ≥3 mm), and lobar location. ③Ablation characteristics: ablation power and time, number of antennas. The minimal ablative safety margin was the key procedural variable of interest. It was measured on the 24-hour post-MWA CT scan by calculating the shortest distance from the edge of the ablation zone (defined by ground-glass opacity) to the edge of the original tumor in all three orthogonal planes, with the final value representing the smallest measured margin. ④Peripheral blood immune parameters: Results from pre-MWA, within one-week post-MWA, and at one-month post-MWA tests were recorded. These included T cell subsets (CD3+, CD4+, CD8+), and plasma cytokine levels (Interleukin-2 [IL-2], IL-6, IL-10, interferon-gamma [IFN-γ], tumor necrosis factor-alpha [TNF-α]). These parameters were retrospectively collected from standardized clinical laboratory tests that were performed as part of the patients’ peri-procedural care.

### 2.4 Statistical analysis

Statistical analyses were performed using SPSS software. Continuous variables are presented as mean ± standard deviation (SD) if normally distributed, or as median and interquartile range (IQR) if non-normally distributed. They were compared using the Student’s t-test or the Mann–Whitney U test, as appropriate. Categorical variables are presented as frequency (percentage) and compared using the χ² test or Fisher’s exact test. Univariate and multivariate logistic regression analyses were used to identify independent risk factors for early LTP. Generalized estimating equations (GEE) were used to analyze the interaction effect between “group” and “time point” on immune parameters, assessing differences in dynamic immune trends between groups. A P-value < 0.05 was considered statistically significant.

## 3 Results

### 3.1 Patient baseline characteristics

**Figure 1:**
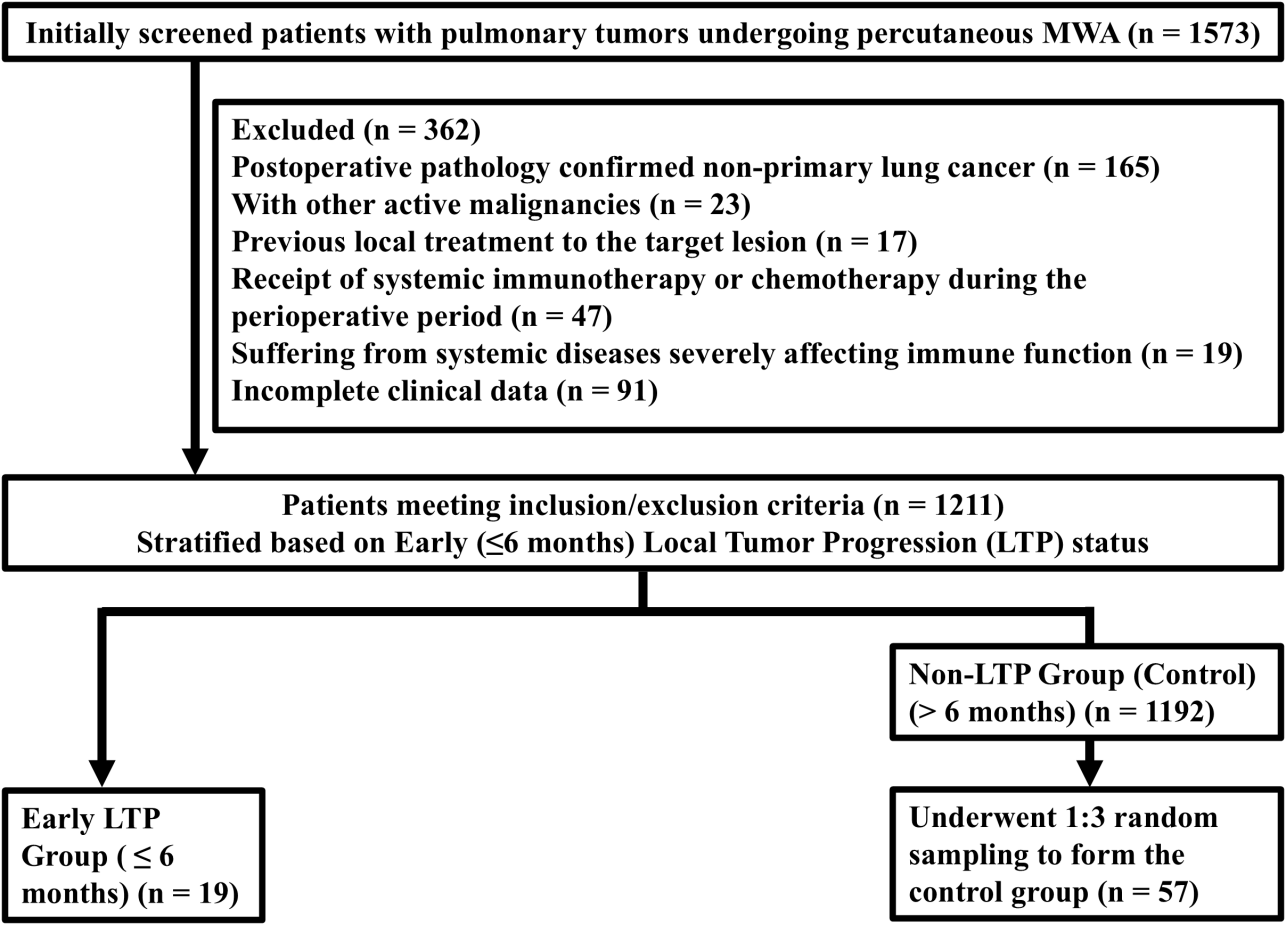
Flowchart of patient selection.

**Figure 1** shows the study participant selection flowchart. A total of 1,573 patients who underwent CT-guided percutaneous pulmonary MWA between January 1, 2021, and December 31, 2023, were initially screened. After applying the inclusion and exclusion criteria, 362 patients were excluded for various reasons, resulting in 1,211 eligible patients with NSCLC. From this cohort, 76 patients were included in the final analysis (19 in the early LTP group, 57 in the control group). **Table 1** presents the baseline characteristics of the two groups. No significant differences were observed in age, sex, ECOG score, or pathological type, indicating comparability. Compared with the control group, the early LTP group had a significantly larger maximum tumor diameter (28.95 ± 7.05 mm vs. 19.25 ± 4.34 mm), a higher proportion of tumors located ≤10 mm from the hilum (78.9% vs. 14.0%), a significantly smaller ablative safety margin (5.0 mm, IQR 4.0–7.0 mm vs. 12.0 mm, IQR 11.0–14.0 mm, P < 0.0001), and a higher proportion of patients with an ablative safety margin ≤ 5 mm (73.68% vs. 17.54%) (P < 0.0001). There were no significant differences in lobar location (P = 0.750) or number of antennas used (P = 0.591) between the two groups.

**Table 1:** Comparison of baseline characteristics between the early LTP group and the control group.

Characteristic	Control group (n = 57)	Early LTP group (n = 19)	p-value
Age (years), Mean ± SD	64.09 ± 4.17	65.32 ± 4.89	0.291
Gender, Male, n (%)	29 (50.88%)	11 (57.89%)	0.592
ECOG Score ≥2, n (%)	10 (17.54%)	6 (31.58%)	0.183
Pathology, Squamous, n (%)	9 (15.79%)	3 (15.79%)	>0.999
Max Tumor Diameter (mm), Mean ± SD	19.25 ± 4.34	28.95 ± 7.05	**<0.0001**
Max Tumor Diameter **>** 30 mm, n (%)	5 (8.77%)	11 (57.89%)	**<0.0001**
Distance to Hilum (mm), Mean ± SD	19.98 ± 5.30	6.47 ± 3.59	**<0.0001**
Distance to Hilum ≤10 mm, n (%)	8 (14.04%)	15 (78.95%)	**<0.0001**
Distance to Major Blood Vessel ≤ 10 mm, n (%)	12 (21.05%)	7 (36.84%)	0.222
Tumor Density, Subsolid, n (%)	21 (36.84%)	3 (15.79%)	0.0841
Lobar location, n (%)			0.750
Left upper lobe, n (%)	18 (31.58)	7 (36.84)	
Left lower lobe, n (%)	8 (14.04)	2 (10.53)	
Right upper lobe, n (%)	19 (33.33)	8 (42.11)	
Right middle lobe, n (%)	2 (3.51)	1 (5.26)	
Right lower lobe, n (%)	10 (17.54)	1 (5.26)	
Number of antennas used			0.591
Single antenna, n (%)	23 (40.35)	6 (31.58)	
Two antennas, n (%)	34 (59.65)	13 (68.42)	
Ablative Safety Margin, mm Mean (Q1, Q3)	12.0 (11.0, 14.0)	5.0 (4.0, 7.0)	**<0.0001**
Ablative Safety Margin ≤ 5mm, n (%)	10 (17.54%)	14 (73.68%)	**<0.0001**

SD, Standard Deviation; ECOG, Eastern Cooperative Oncology Group; IQR, Interquartile Range. Data are presented as mean ± SD, n (%), or median (IQR). P-values were calculated using Student’s t-test for normally distributed continuous variables, Mann-Whitney U test for non-normally distributed continuous variables, and Chi-square test (or Fisher’s exact test where appropriate) for categorical variables. Statistically significant P-values (P < 0.05) are highlighted in bold.

### 3.2 Risk factor analysis for early LTP after MWA

**Figure 2:**
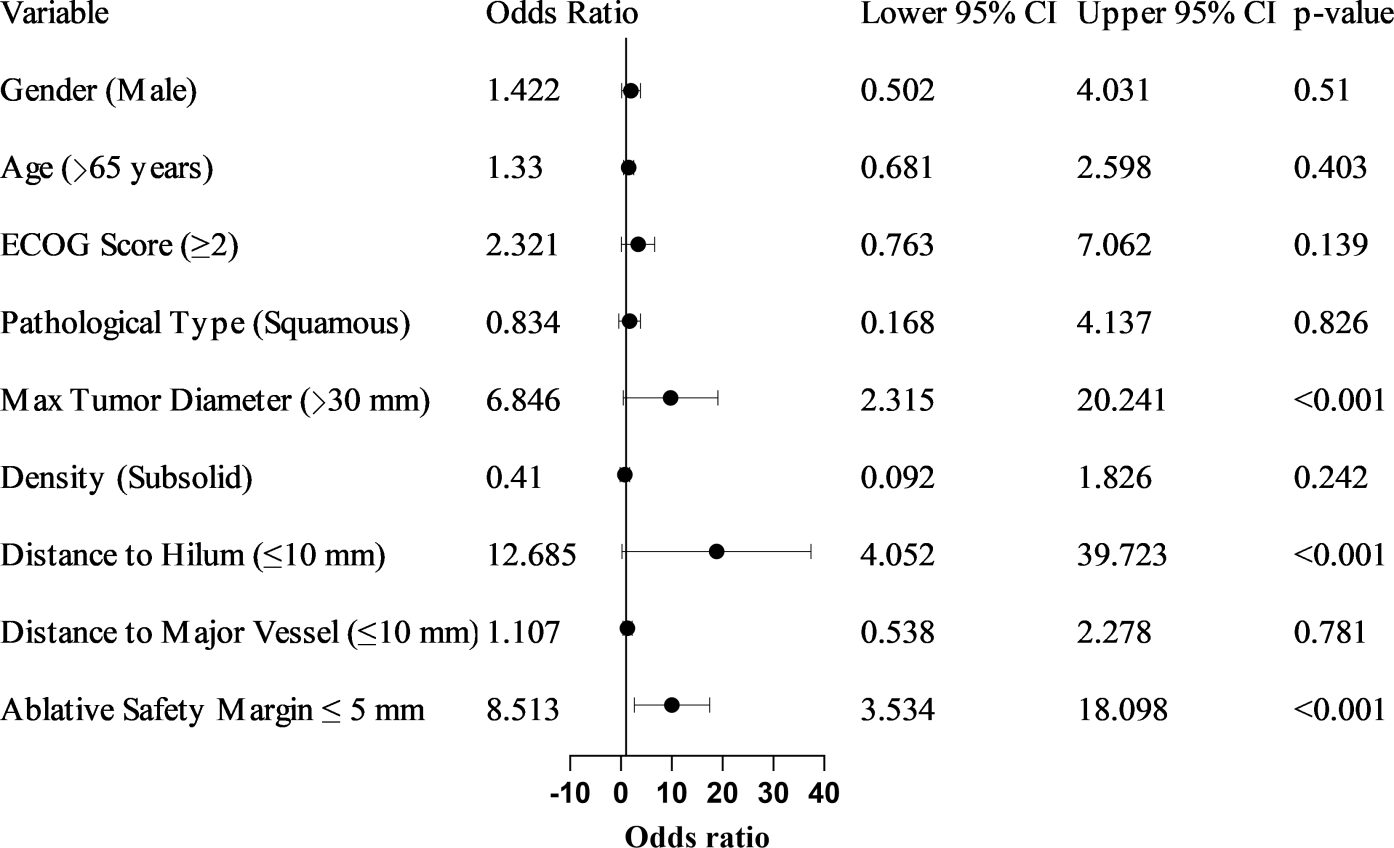
Forest plot of univariate logistic regression analysis for factors associated with early local tumor progression post-MWA. Odds ratios (OR) with 95% confidence intervals (CIs) are shown for each variable. The vertical line at OR = 1 indicates the null value. Three factors—maximum tumor diameter >30 mm, distance to hilum ≤ 10 mm, and ablative safety margin ≤ 5 mm —were identified as significant risk factors.

Univariate logistic regression analysis (**Table 2**, **Figure 2**) identified maximum tumor diameter >30 mm, distance to the hilum ≤10 mm, and an ablative safety margin ≤ 5 mm as significant risk factors, with odds ratios (OR) of 6.846 (95% CI: 2.315–20.241), 12.685 (95% CI: 4.052–39.723), and 8.513 (95% CI: 3.534–18.098), respectively. In the multivariate logistic regression analysis (**Table 3**), maximum tumor diameter >30 mm (OR = 2.681, 95% CI: 1.218–5.901, P = 0.014), distance to the hilum ≤10 mm (OR = 3.280, 95% CI: 1.678–6.411, P = 0.001), and ablative safety margin ≤ 5 mm (OR = 4.152, 95% CI: 1.922–8.968, P < 0.001) remained independent predictors.

**Table 2:** Univariate logistic regression analysis for early LTP post-MWA.

Variable	β value	OR	95% CI	p-value
Gender (Male)	0.352	1.422	0.502–4.031	0.510
Age (>65 years)	0.285	1.330	0.681–2.598	0.403
ECOG Score ≥2	0.842	2.321	0.763–7.062	0.139
Pathology Type (Squamous)	−0.182	0.834	0.168–4.137	0.826
Max Tumor Diameter >30 mm	1.924	6.846	2.315–20.241	<0.001
Tumor Density (Subsolid)	-0.892	0.410	0.092–1.826	0.242
Distance to Hilum ≤ 10 mm	2.541	12.685	4.052–39.723	<0.001
Distance to Major Vessel ≤10 mm	0.102	1.107	0.538–2.278	0.781
Ablative Safety Margin ≤ 5 mm	2.35	8.513	3.534–18.098	< 0.001

OR, Odds Ratio; CI, Confidence Interval; ECOG, Eastern Cooperative Oncology Group. The reference category for each categorical variable is listed second in the parentheses.

**Table 3:** Multivariate logistic regression analysis for early LTP post-MWA.

Variable	β value	OR	95% CI	P-value
Max Tumor Diameter >30 mm	0.986	2.681	1.218–5.901	0.014
Distance to Hilum ≤ 10 mm	1.188	3.280	1.678–6.411	0.001
Ablative Safety Margin ≤ 5mm	1.855	4.152	1.922–8.968	< 0.001

OR, Odds Ratio; CI, Confidence Interval. The model included all variables with P < 0.10 in the univariate analysis. The reference category for each variable is listed second in the parentheses.

### 3.3 Dynamic differences in peripheral blood immune profiles

**Figure 3:**
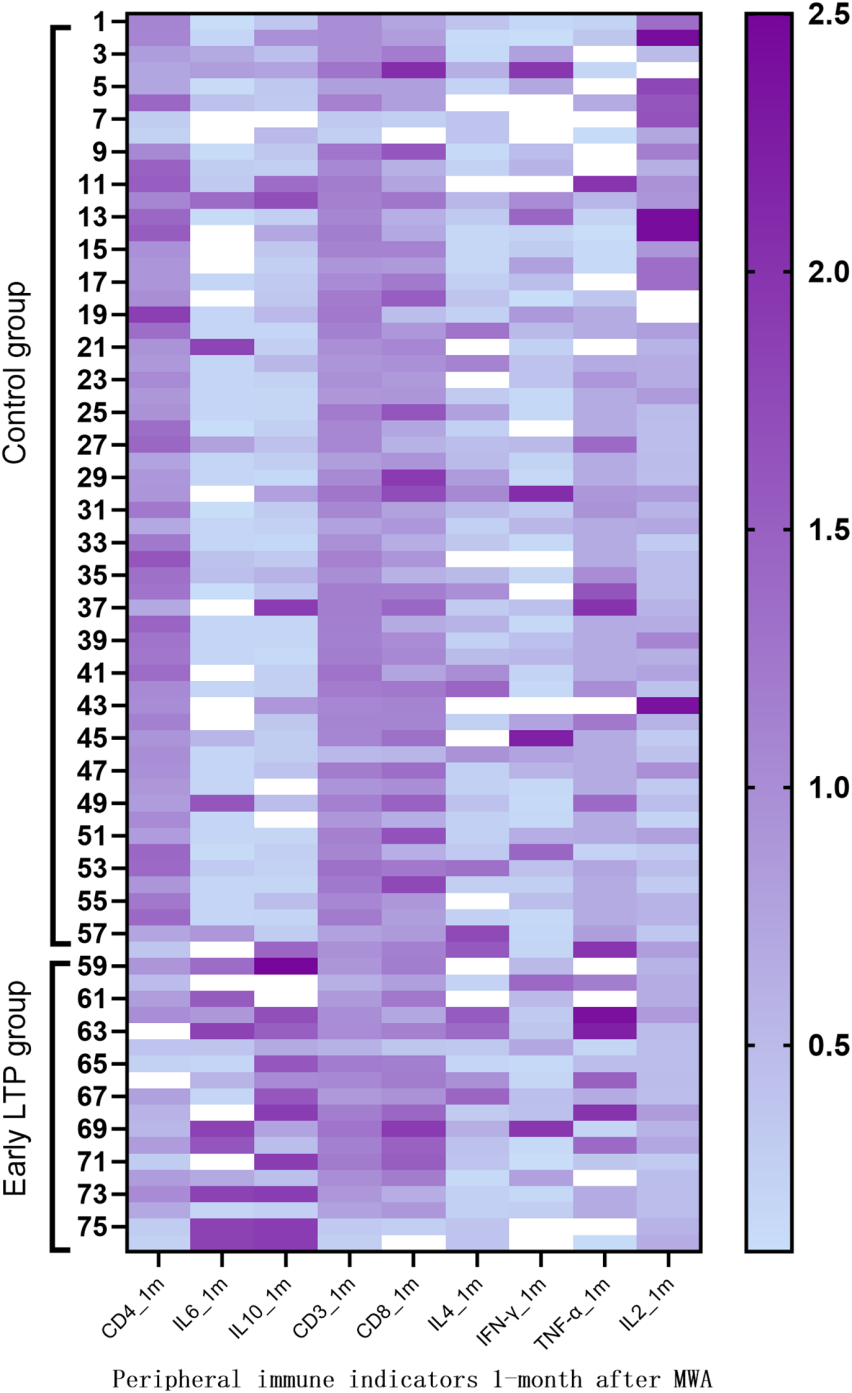
Systemic immune landscape at one-month post-MWA. The heatmap depicts normalized immune profiles for Control (n = 57) and Early LTP (n = 19) groups. Values are normalized to the mean of each parameter within its respective group (mean = 1). Each row represents an individual patient, and each column an immune parameter. Deep purple indicates values above the group mean, and light purple values below the group mean.

**Figure 4:**
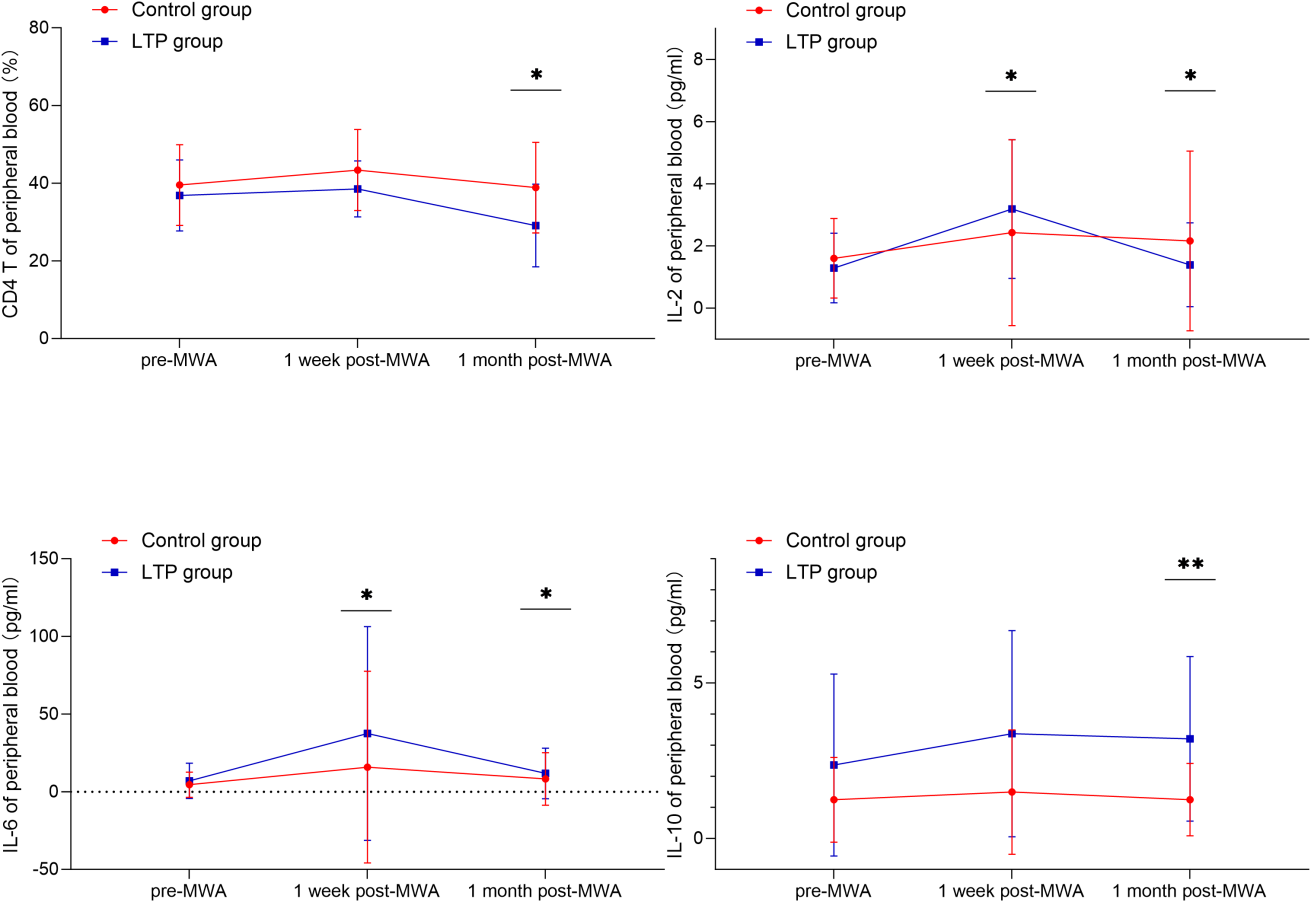
Longitudinal trends of selected peripheral blood T cell subsets (CD4^+^ T cells) and cytokines (IL-2, IL-6, IL-10) at three time points: pre-MWA, 1-week post-MWA, and 1-month post-MWA. The Early LTP group and the Control group are represented by a solid blue line and a solid red line, respectively. Data are presented as mean ± standard deviation. Statistical comparisons between groups at each time point are indicated as follows: *p < 0.05; **p < 0.01; ***p < 0.001.

**Table 4** shows a comparative analysis of peripheral blood immune parameters between the two groups of patients. At one-week post-MWA, both groups showed early systemic inflammatory response. Compared with the control group, LTP group significantly increased levels of IL - 2 (median: 3.20 pg/mL vs. 2.35 pg/mL, P = 0.029), IL - 6 levels also significantly elevated (median: 8.16 pg/mL vs. 2.08 pg/mL, P = 0.037). Compared with the control group at a month post-MWA, the proportion of CD4^+^ T cells in the peripheral blood of the early LTP group was significantly lower (median: 32.50% vs. 33.96%, P = 0.040), the level of IL-10 was higher (median: 4.10 pg/ml vs. 1.00 pg/ml, P < 0.001), the level of IL-6 also increased (median: 6.70 pg/ml vs. 2.08 pg/ml, P = 0.004), the level of IL-2 was significantly lower (median: 1.48 pg/ml vs. 2.35 pg/ml, P = 0.020). At any point in time, other parameters between the two groups were no statistically significant difference, including CD3 ^+^ and CD8 ^+^ in T cell percentage and IFN-γ, IL - 4 and TNF-α levels. **Figure 3** shows the heatmap visualization of the peripheral blood immune landscape at one-month post-MWA. The heatmap displays normalized expression levels of key immune parameters for each patient in the Control group (n=57, left) and the Early LTP group (n=19, right) at the one-month follow-up time point. **Figure 4** shows the dynamic changes of some peripheral blood immune parameters perioperative period.

**Table 4:** Comparison of peripheral blood immune parameters between control group and early LTP group perioperative period.

Parameter	Time point	Control group (n = 57)	Early LTP group (n = 19)	p-value
CD3+ (%)	Pre-MWA	68.14 (62.05, 73.90)	68.50 (62.22, 76.68)	0.975
	1 Week post-MWA	70.47 (62.38, 77.57)	67.13 (62.56, 70.10)	0.148
	1 Month post-MWA	70.81 (59.78, 78.47)	63.80 (56.50, 76.74)	0.344
CD4+ (%)	Pre-MWA	38.72 (33.88, 47.67)	40.54 (35.07, 45.52)	0.853
	1 Week post-MWA	40.16 (34.76, 49.89)	37.39 (34.76, 41.98)	0.227
	1 Month post-MWA	33.96 (30.17, 44.63)	32.50 (25.20, 37.20)	**0.040**
CD8+ (%)	Pre-MWA	24.52 (18.47, 32.69)	26.30 (17.33, 33.77)	0.871
	1 Week post-MWA	22.70 (16.99, 29.61)	28.20 (21.36, 29.80)	0.285
	1 Month post-MWA	23.33 (17.28, 31.74)	24.50 (22.08, 32.80)	0.146
IFN-γ (pg/mL)	Pre-MWA	2.23 (1.89, 8.65)	4.57 (2.13, 6.80)	0.333
	1 Week post-MWA	2.23 (1.89, 8.95)	4.10 (1.91, 6.10)	0.629
	1 Month post-MWA	4.54 (1.91, 22.40)	4.10 (2.10, 8.95)	0.608
IL-2 (pg/mL)	Pre-MWA	1.48 (0.78, 2.35)	1.75 (1.12, 2.24)	0.508
	1 Week post-MWA	2.35 (1.20, 4.50)	3.20 (1.84, 5.35)	**0.029**
	1 Month post-MWA	2.35 (1.84, 4.26)	1.48 (0.94, 2.35)	**0.020**
IL-10 (pg/mL)	Pre-MWA	0.89 (0.53, 1.81)	0.90 (0.53, 4.70)	0.509
	1 Week post-MWA	0.93 (0.64, 2.68)	1.25 (0.74, 5.40)	0.349
	1 Month post-MWA	1.00 (0.59, 2.51)	4.10 (1.93, 7.10)	**<0.001**
IL-4 (pg/mL)	Pre-MWA	1.71 (0.60, 1.95)	1.63 (0.71, 8.30)	0.442
	1 Week post-MWA	0.86 (0.71, 2.35)	1.44 (0.71, 45.10)	0.231
	1 Month post-MWA	2.39 (0.71, 4.09)	2.90 (1.08, 8.10)	0.431
IL-6 (pg/mL)	Pre-MWA	2.08 (1.75, 8.09)	1.75 (1.75, 11.70)	0.868
	1 Week post-MWA	2.08 (1.75, 8.09)	8.16 (1.75, 55.70)	**0.037**
	1 Month post-MWA	2.08 (1.75, 12.50)	6.70 (4.10, 14.10)	**0.004**
TNF-α (pg/mL)	Pre-MWA	2.26 (1.33, 2.26)	2.26 (1.33, 10.32)	0.118
	1 Week post-MWA	2.26 (1.33, 2.75)	2.45 (1.33, 15.80)	0.110
	1 Month post-MWA	5.26 (3.33, 6.17)	6.70 (4.00, 12.40)	0.237

Data is presented as median (interquartile range). MWA, microwave ablation; IFN-γ, interferon-gamma; IL, interleukin; TNF-α, tumor necrosis factor-alpha. Between-group comparisons at each time point were performed using the Mann-Whitney U test. Statistically significant P-values (P < 0.05) are highlighted in bold.

## 4 Discussion

This retrospective nested case-control study is the first to correlate clinical risk factors for iMWA after lung MWA with changes in systemic immune characteristics, providing preliminary evidence that may link iMWA to a state of systemic immune dysregulation characterized by features of immunosuppression and chronic inflammation. First, we confirmed that tumor size, location, and ablation margin are key determinants of local efficacy. Maximum tumor diameter (≥30 mm) and proximity to the hilum (distance ≤10 mm) directly increase the technical difficulty of achieving complete ablation. The “heat-sink” effect from major blood vessels and the complex anatomy of the hilar region may cause uneven heat distribution, creating ablation “dead zones” (9, 21). Our analysis also identified an insufficient ablative safety margin (≤5 mm) as an independent risk factor, which provides a more clinically direct and feasible measure than the ablation volume index (AVI). The control group exhibited a significantly larger median safety margin (12.0 mm) compared to the early LTP group (5.0 mm). This empirical finding aligns with the geometric principle that an inadequate safety margin predicts a high risk of LTP. It is noteworthy that the odds ratio for “distance to hilum ≤10 mm” is presented with a wide confidence interval, which reflects the inherent limitation of our sample size. Nevertheless, the lower bound of the confidence interval being >1 consistently indicates that perihilar location is a significant and robust technical risk indicator for early LTP. It is noteworthy that the previously reported AVI threshold (≤2.2) corresponds to a safety margin of approximately 5–10 mm, which is consistent with our observed margin-based risk threshold (22). In addressing these technical challenges, recent research has explored the use of auxiliary planning tools, such as the three-dimensional visualization planning system (3D-VAPS) described by Hu et al. for guiding microwave ablation in early-stage NSCLC, demonstrating its potential to enhance procedural safety and feasibility (23). Although achieving safety ablation margins is the most important technical goal, in some clinical challenging situations - such as for larger tumors or those located near crisis structures (such as the hilum or major blood vessels) - achieving complete technical ablation may be limited, thereby making iMWA a potential therapeutic outcome. At this point, the urgent task in clinical practice is to actively monitor and manage the risks and biological consequences associated with these situations, and our research aims to preliminarily explore these related factors.

**Figure 5:**
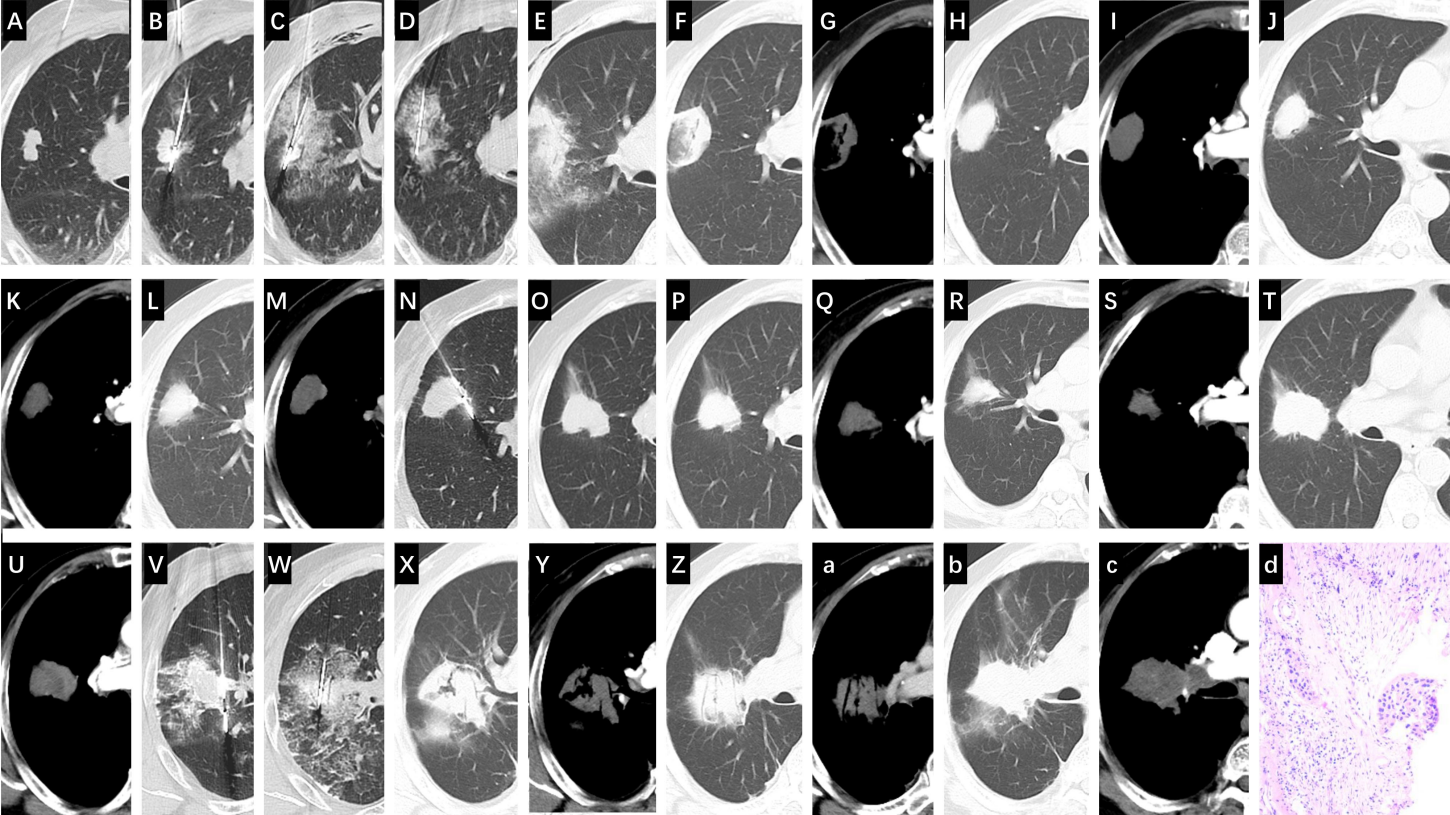
Illustrative Case of Late Local Tumor Progression Managed with Serial microwave ablation (MWA) for an stage IB (AJCC 8th edition) primary lung squamous cell carcinoma. **(A)** Pre-MWA CT scan showing a 21 mm × 33 mm mass in the right upper lobe. **(B, C)** First MWA session: positioning of two antennas at the proximal and distal ends of the tumor. **(D)** Concomitant core needle biopsy performed during the first MWA session, confirming the diagnosis of squamous cell carcinoma. **(E)** 24-hour post-MWA CT scan showing the ablation zone completely encompassing the tumor. Initial Follow-up (Months 1-10): **(F, G)** One-month follow-up CT scan showing a stable, non-enhancing ablation zone. **(H, I)** Four-month follow-up CT scan showing a stable, non-enhancing ablation zone. **(J, K)** Ten-month follow-up CT scan showing a stable, non-enhancing ablation zone. First Local Tumor Progression & Second MWA (Month 14): **(L, M)** Fourteen-month follow-up CT scan revealing enlargement and enhancement at the proximal margin (arrows), consistent with local tumor progression (LTP). **(N)** Second MWA session: positioning of a single antenna at the site of LTP. Follow-up after Second MWA (Months 15-20): **(O–Q)** Follow-up CT scans at months 15 and 16 showing the ablation zone with no enhancement. **(R, S)** Follow-up CT scan at month 18 showing a stable, non-enhancing ablation zone. **(T, U)** Follow-up CT scan at month 20 demonstrating new enhancement at the proximal margin, indicative of residual tumor. Third MWA & Final Follow-up (Months 21-27): **(V, W)** Third MWA session (performed at month 21): positioning of two antennas to cover the residual tumor. **(X, Y)** Follow-up CT scan at month 22 (one-month post-third MWA) showing a satisfactory ablation zone without enhancement. **(Z, a)** Follow-up CT scan at month 24 showing a stable, non-enhancing zone. **(b–d)** Follow-up CT scan at month 27 showing a stable, non-enhancing ablation zone, indicating no evidence of LTP at this follow-up interval. The initial biopsy revealed squamous cell carcinoma (p40+, p63+, CK7-).

The main, novel finding of this study lies in the comprehensive immunological profile we detected. We observed that in the early phase post-MWA, a similar acute inflammatory response was triggered regardless of ablation completeness, reflecting non-specific immune activation related to tissue injury. However, the long-term immune trajectory diverged between groups. Building on our data, we have moved beyond a static description of “immunosuppression”. Instead, we now frame our findings as evidence of a temporal trajectory of immune dysregulation following ablation. In contrast to patients with complete ablation, patients with early LTP exhibited features of profound and multifaceted immunosuppression combined with chronic inflammation at one-month after the procedure. The significant decline in CD4^+^ T cells and the increase in immunosuppressive cytokines, paints a picture of suppressed adaptive and innate immunity. This is consistent with the conclusions from previous studies (24–28). This phenomenon strongly suggests that residual tumor cells may actively shape and maintain a suppressive systemic immune environment. Persistent tumor antigen exposure and inflammatory stimulation might drive compensatory immunosuppressive mechanisms, among which the sustained IL-10 elevation is central for counteracting potential autoimmune damage and suppressing anti-tumor immunity (29–31). The concurrent significant rise in IL-6 indicates a state of unresolved inflammation, known to promote tumor progression and fuel immunosuppressive networks. Interestingly, the transiently elevated IL-2 levels observed at one week in the early LTP group may reflect an initial phase of T cell activation, but in the context of high IL-10, this signal may be co-opted to promote immunosuppressive pathways rather than effective effector T cell responses (31–33). It should be noted that, as a retrospective study, our analysis did not include specific measurements of regulatory T cells (Tregs) or natural killer (NK) cells, or more comprehensive systemic inflammatory indices (e.g., platelet count, neutrophil-to-lymphocyte ratio). However, existing literature strongly indicates that alterations in Tregs (e.g., expansion) and NK cells (e.g., functional impairment) are important indicators of immune dysfunction in cancer patients and are often associated with tumor progression and poor prognosis (34, 35). Future prospective studies incorporating these cellular subsets would provide a more complete picture of the immune landscape following MWA. The dual and complex impact of MWA on the immune system—initial activation followed by potential suppression or dysregulation—prompts the exploration of its combination with systemic therapy for synergistic effects as supported by previous studies. This understanding potentially opens a novel therapeutic paradigm for exploration. The observed time-dependent immune changes suggest the existence of critical post-procedural windows (e.g., the one-month time point in our study). Within such windows, monitoring deviations in specific immune parameters could potentially serve as dynamic biomarkers for the early identification of patients at elevated risk for progression, which might help guide more intensive surveillance schedules. More speculatively, the identified window might represent a candidate moment for adjuvant immunomodulation. Future strategies could explore combining ablation with immune checkpoint inhibitors or agents targeting specific inflammatory pathways (e.g., IL-6/IL-10), with the aim of intercepting and reprogramming a nascent pro-tumorigenic milieu. This exploratory concept could potentially transform the challenge of iMWA into a strategic opportunity for synergistic combination therapy to prevent LTP, and is tentatively supported by previous studies showing promise for MWA combined with systemic therapies (36–39). For patients who nonetheless develop early LTP, timely salvage therapy remains a viable option based on current practice. Our clinical experience and existing literature indicate that repeat MWA can be an effective strategy in these cases. **Figure 5** shows the imaging timeline of a representative patient with stage IB lung squamous cell carcinoma managed with serial MWA, demonstrating LTP at 14 and 20 months and subsequent successful salvage ablation with sustained local control at 27-month follow-up.

This study has several limitations. The single-center retrospective design, small sample size, and sole reliance on peripheral blood markers limit the generalizability of our findings and preclude in-depth analysis of the local tumor microenvironment. Critically, the lack of an untreated control group with comparable high-risk tumors makes it impossible to clarify whether the observed immunosuppressive state is induced by incomplete ablation or is inherent to larger tumor biology. Furthermore, while inadequate ablation margins near the hilum are strongly associated with recurrence, the contribution of intrinsic tumor biology in this location cannot be excluded. Additionally, the analysis focused on a selected panel of T-cell subsets and cytokines and did not include more comprehensive systemic inflammatory indices. Moreover, the definition of early LTP in this study was based on pragmatic imaging and temporal criterion (≤6 months) and did not incorporate more complex biological or molecular markers. As highlighted by Bonis et al., the classification of recurrence patterns in lung cancer urgently requires standardization. Future prospective studies should aim to establish and validate a standardized LTP definition system for the thermal ablation field, integrating imaging, pathological, and molecular features (40). Future prospective, multi-center studies incorporating tissue-based analyses and appropriate control cohorts are needed to validate these findings and precisely delineate the immunological impact of iMWA.

These findings, albeit derived from a limited cohort, suggest that LTP after MWA in patients with NSCLC is closely associated with anatomical tumor factors and systemic immune dysregulation in the host. In the future, optimizing preprocedural MWA plans to use precise planning technologies like 3D-VAPS with the explicit goal of achieving a sufficient ablative safety margin and actively exploring combined postoperative immunomodulatory strategies for high-risk patients may be key to preventing LTP and improving treatment efficacy.

## Data Availability

The raw data supporting the conclusions of this article will be made available by the authors, without undue reservation.
